# Supplementary material for: Content-rich biological network constructed by mining PubMed abstracts
Source: BMC Bioinformatics. 2004 Oct 8;5:147. doi: 10.1186/1471-2105-5-147 (PMC528731; doi:10.1186/1471-2105-5-147)
Supplement: Additional File 2 — The original results of the above study (non-essential files are deleted to keep the file size under the limit set by BMC bioinformatics). [file 1471-2105-5-147-S2.bz2 › chilibotAdditionalFile2/dip05/158ID7685215E560/html/FOS_TBP.html]

 


 **FOS** and **TBP** 
  
Found 30 abstracts in PubMed, retrieved 05.  
 

 What does Google say? 
 PDF only 
| .edu only 

---

**Interactive relationship** (e.g. stimulation, inhibition, etc)

**Non-interactive relationship** (e.g. studied together, co-existance, homology, etc.)

- Mutation of either Tyr248, Tyr357, or Tyr462 to phenylalanine reduced transcription from a c  **fos**  promoter relative to wild type BAP  [ **TBP** ]  TFII I in transfected COS 7 cells, consistent with the interpretation that phosphorylation at these sites contributes to transcriptional activation.  Ref: 11373296 J Biol Chem, 2001
- Strikingly, the first modification observed is methylation of histone H3 lysine 4, which correlates with the binding of the SET9 methyltransferase and the assembly of a complex consisting of c Jun, c  **Fos** , TATA binding protein  [ **TBP** ] , and RNA polymerase II.  Ref: 12588998 Mol Cell Biol, 2003
- These includes nuclear factor,  **FOS**  related antigen 1, cytoplasmic dynein light chain, replication factor C, nucleoside diphosphate kinase B, UV excision repair protein, insulin like growth factor receptor II, transcription initiation factor TFIID  [ **TBP** ]  subunit, growth factor receptor bound protein 2, UV excision repair protein, glutathione peroxidase, Y box binding protein 1 and heat shock protein 86.  Ref: 10946852 Life Sci, 2000
